# Competing at the Cybathlon championship for people with disabilities: long-term motor imagery brain–computer interface training of a cybathlete who has tetraplegia

**DOI:** 10.1186/s12984-022-01073-9

**Published:** 2022-09-06

**Authors:** Attila Korik, Karl McCreadie, Niall McShane, Naomi Du Bois, Massoud Khodadadzadeh, Jacqui Stow, Jacinta McElligott, Áine Carroll, Damien Coyle

**Affiliations:** 1 Intelligent Systems Research Centre, Ulster University, Derry, UK; 2 National Rehabilitation Hospital of Ireland, Dun Laoghaire, Ireland; 3 University College Dublin, Dublin, Ireland

**Keywords:** Brain–computer interface (BCI), Motor imagery, Electroencephalography (EEG), Competition, Tetraplegia, Long-term training, Neurofeedback, Neurogaming, Alternative and augmentative and assistive communication (AAC) device

## Abstract

**Background:**

The brain–computer interface (BCI) race at the Cybathlon championship, for people with disabilities, challenges teams (BCI researchers, developers and pilots with spinal cord injury) to control an avatar on a virtual racetrack without movement. Here we describe the training regime and results of the Ulster University BCI Team pilot who has tetraplegia and was trained to use an electroencephalography (EEG)-based BCI intermittently over 10 years, to compete in three Cybathlon events.

**Methods:**

A multi-class, multiple binary classifier framework was used to decode three kinesthetically imagined movements (motor imagery of left arm, right arm, and feet), and relaxed state. Three game paradigms were used for training i.e., NeuroSensi, Triad, and Cybathlon Race: BrainDriver. An evaluation of the pilot’s performance is presented for two Cybathlon competition training periods—spanning 20 sessions over 5 weeks prior to the 2019 competition, and 25 sessions over 5 weeks in the run up to the 2020 competition.

**Results:**

Having participated in BCI training in 2009 and competed in Cybathlon 2016, the experienced pilot achieved high two-class accuracy on all class pairs when training began in 2019 (decoding accuracy > 90%, resulting in efficient NeuroSensi and Triad game control). The BrainDriver performance (i.e., Cybathlon race completion time) improved significantly during the training period, leading up to the competition day, ranging from 274–156 s (255 ± 24 s to 191 ± 14 s mean ± std), over 17 days (10 sessions) in 2019, and from 230–168 s (214 ± 14 s to 181 ± 4 s), over 18 days (13 sessions) in 2020. However, on both competition occasions, towards the race date, the performance deteriorated significantly.

**Conclusions:**

The training regime and framework applied were highly effective in achieving competitive race completion times. The BCI framework did not cope with significant deviation in electroencephalography (EEG) observed in the sessions occurring shortly before and during the race day. Changes in cognitive state as a result of stress, arousal level, and fatigue, associated with the competition challenge and performance pressure, were likely contributing factors to the non-stationary effects that resulted in the BCI and pilot achieving suboptimal performance on race day.

*Trial registration* not registered

**Supplementary Information:**

The online version contains supplementary material available at 10.1186/s12984-022-01073-9.

## Background

The Cybathlon championship is a unique competition in which people with physical disabilities compete against each other to complete tasks and challenges using state-of-the-art technical assistance systems. The event serves as a platform for technology developers to exchange ideas and collaborate closely with people with physical disabilities as they develop their devices—Cybathlon aims to drive research on assistance systems for everyday use, and promote public dialogue [1, 2].

The Cybathlon brain–computer interface (BCI) event challenges teams to control a virtual race vehicle (avatar) on a virtual race track (video game platform). The majority of teams involved in the BCI race at the 2016 Cybathlon championship (prior to the events reported in this paper), focused on training pilots to modulate brain rhythms using a motor imagery based BCI to control the avatar [3]. This study documents the Ulster University (NeuroCONCISE) team’s training and control strategy involving a 4-class motor imagery based BCI for Cybathlon race events in 2019 and 2020.

### Motor imagery BCI

A motor imagery BCI requires the user to imagine performing movements or to mentally simulate a physical action without activating any muscular pathways. Extensive research on motor imagery paradigms has explicitly shown that maximal discrimination accuracy is achieved using lateralized differences in mu (8–12 Hz) and beta (12–30 Hz) band power [4–6]. These power changes are linked with event-related (de)synchronization (ERD/ERS) of neural activity in sensorimotor areas and originate from decreased or increased phase-locked synchronous activity of specific neuron populations over cortical motor areas [7]. Lateralized differences in sensorimotor rhythms (SMR) enable discrimination of the imagined movement of different limbs and muscles controlling different parts of the body [8].

Learning intentional, goal-directed modulation of sensorimotor rhythms (from imagined movement) requires practice with sensory feedback [6]. Motor imagery based BCI typically requires training to achieve a reasonably high level of control [9]. To reach this criterion participants generally undergo multiple sessions lasting from one to several hours per day, over days or weeks. Motor imagery based BCI training feedback may be presented using various modalities including somatosensory (via vibrotactile/electrical stimulation), auditory, or visual—though most often using the visual channel, whilst there is increasing interest in the other feedback modalities [10, 11]. The difference between presentation methods is considered to be small, thus enabling the development of more naturalistic feedback paradigms [12] that do not focus on the visual sense.

Motivation has also been shown to be an important aspect of BCI motor imagery performance, especially in BCI target user groups [13]. Therefore, gamification is often employed in an attempt to enhance feedback presentation and hence increase motivation. The Cybathlon BCI race event builds on this and challenges competitors (teams and pilots) to control in real-time, an avatar on a virtual racetrack using a BCI, in front of an audience. A significant challenge is thus presented, requiring the pilot to accurately and repetitively modulate brain states that can be detected by the BCI, associated with three race commands plus a no-control state, i.e., resting or relax state.

### Factors that impact BCI control accuracy achieved over a multi-session BCI training

Perdikis et al. [14], highlighted that a successful BCI application should hang on three pillars: the BCI device, the interaction between the BCI and the user, and the actual application wherein the BCI is used. The BCI device is required to accurately decode imagined commands given by the user. A long-term training period provides an opportunity for both the fitting of the BCI’s hyper-parameters to the user’s voluntary brain activity, and real-time feedback that enables the brain to adapt to the required cognitive state. Thus, the training period can be used to improve performance during a multi-session learning process [15].

Ponferrada and colleagues [16], improved the efficiency of their BCI system so that the amount of training data required to learn real-time control for Cybathlon was reduced, thereby shortening the time required for the training phase. Benaroch et al. [9], combined a progressive multi-class mental task based BCI with a machine learning algorithm that uses adaptive Riemannian classifiers—aimed at improving BCI control by producing electroencephalography (EEG) activity that increasingly matches the BCI classifier, rather than improving the classifier to better discriminate the EEG activity. Turi et al. [17] also reported an improvement in their pilot’s BCI control accuracy during a 2 and half months training performed between mid-June and the end of August in 2019. However they reported a drop in performance during the Cybathlon competition race, compared to performance during training. They suggested that the drop was due to the indirect impact of stress on performance—expressly the user’s psychological state influenced their ability to concentrate, which affected their performance negatively. Hence, variability in the signal between sessions is largely considered to be due to shifts in the user’s psychological state, e.g., due to fatigue or loss of attention [18, 19].

Here we provide details of the BCI setup, training regime, and performance obtained during long-term BCI training of the Ulster University Cybathlon team (NeuroCONCISE) for two race competitions. The findings highlight the challenges in developing a BCI that is capable of adapting to changing user states and environmental conditions that can result in temporal variations in the neural signal and create a barrier to the use of BCI systems in everyday life for individuals with a motor-disability.

## Methods

### Participant

The study involved a single participant (the pilot) who has tetraplegia with normal vision and hearing, aged 49 at the time of the Cybathlon 2020. The pilot suffered a spinal injury (fractured C4–C5) in 1993 during a motorbike accident. Prior to the commencement of the training, the pilot was presented with information regarding the experimental protocol and was asked to read and sign an informed consent form to participate in the study, which was approved by the National Rehabilitation Hospital of Ireland research ethics committee. Before the beginning of the BCI training carried out in 2019 and 2020 (reported in this paper), the pilot took part in 10 basic BCI training sessions in 2009 and 12 training sessions for Cybathlon 2016.

### Experimental paradigms

**Fig. 1:**
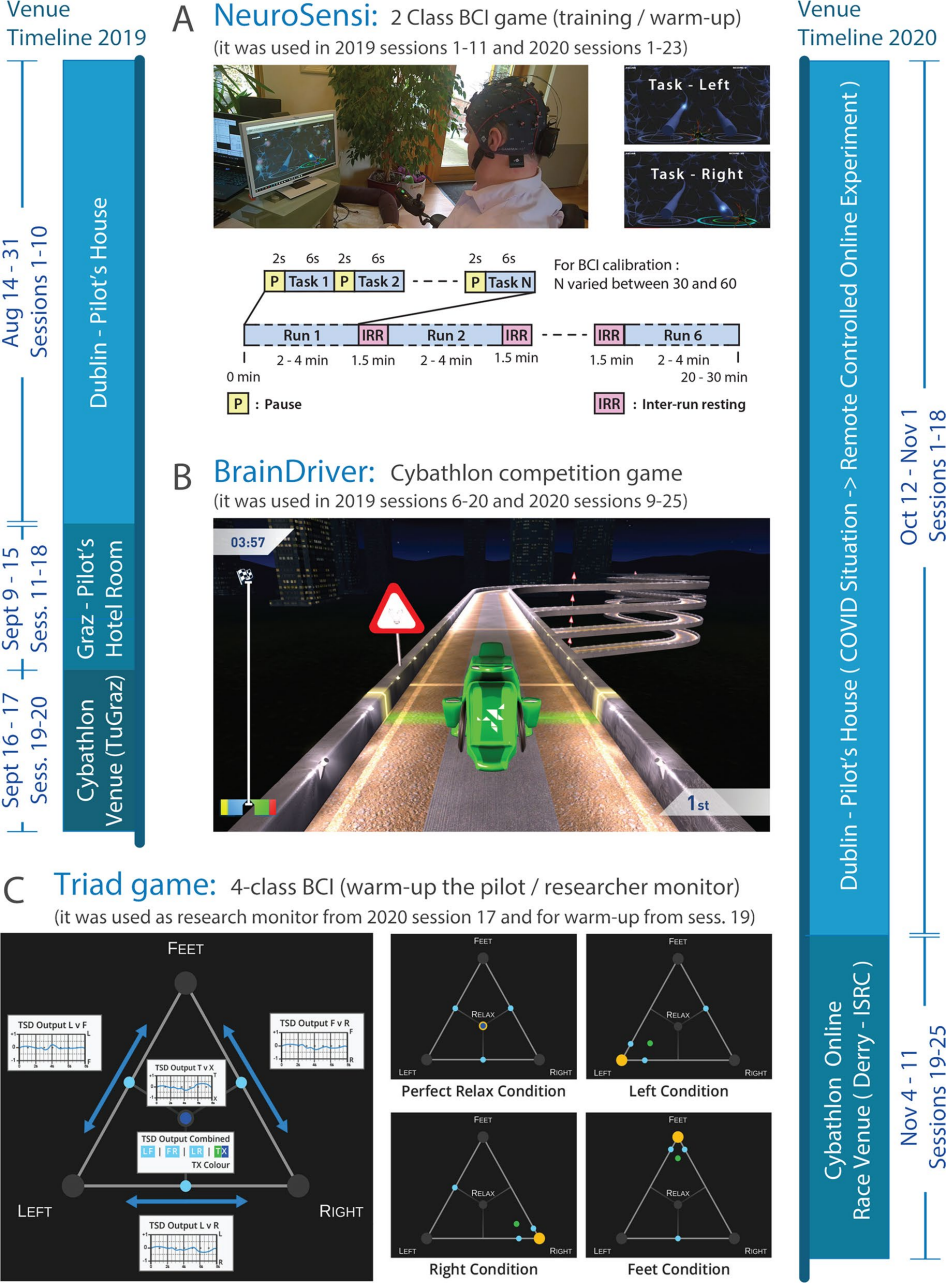
BCI games were used for training our pilot for the Cybathlon BCI event in 2019 and 2020. **A** The NeuroSensi game and the timing of the trials in the 2-class classification based NeuroSensi experiments (Additional file 1: Video S1). **B** The BrainDriver race (Additional file 2: Video S2 and Additional file 3: Video S3). **C** The Triad game for monitoring the multi-class classification results and pilot warm-up, using a linear combination of multiple 2-class classifiers. The 2D position of the ball within the triangle is calculated using the results of the three ‘task vs. task’ 3-class classifiers (LF, LR, FR). The colour of the ball within the triangle is controlled by the ‘task vs. relax’ (TX) classifier (blue: task (T), green: relax(R)) (Additional file 4: Video S4). The venue timelines show time periods along with session intervals when the pilot was trained in different locations for completing the Cybathlon BCI series in 2019 and the Cybathlon Global Edition in 2020

The BrainDriver BCI racing game was used in the Cybathlon BCI challenge (described at the end of this section). Our online BCI uses analogue outputs of four 2-class classifiers to relay control commands to the BrainDriver game (described in “The online BCI” section). The BrainDriver race is controlled using commands received from the BCI but does not provide continuous feedback to the pilot about the analogue output of four 2-class classifiers which are built into the BCI framework. As accurate control of the avatar in the BrainDriver race requires each of the four 2-class classifiers to be pilot-specifically calibrated, data must be collected to train the 2-class classifiers. For this purpose we used another BCI game called NeuroSensi [20, 21]. Additionally, to present the pilot with outputs from four classifiers simultaneously we used a novel paradigm referred to as the Triad game, which is introduced in this study for the first time. These are described below and shown in Fig. 1.

#### NeuroSensi game training for paired motor imagery tasks

The first phase of the BCI training which took place in 2019 and 2020, involved the NeuroSensi BCI game (Fig. 1A, Additional file 1: Video S1) which is played using two motor imagery commands. The NeuroSensi game has a representation of a neuronal axon on both sides of the monitor. Two seconds after the beginning of the trial, a light (representing a neural spike) appears at the beginning of one of the two axons to cue the participant to begin the corresponding motor imagery task. The light takes 6 s to travel over the ‘axon’ during the task period (Fig. 1A). In each NeuroSensi session, six runs were completed wherein different binary combinations of the three commands (left hand (L), feet (F), right hand (R)), and relax (X) were performed. The number of trials in each run acquired for BCI calibration (i.e., for calibrating/training hyper parameters of the BCI framework—described in “Calibration of the two-class classification modules” section) varied between 30–60 (equal number/class), depending on the actual session ID (more trials in the initial session, fewer trials in later sessions). The time duration of a run, therefore, varied between 240 and 480 s. The time duration of six runs involving L vs. R (LR), FR, LF, LX, FX, and XR tasks, including five 90 s inter-run pauses, varied between 20 and 30 min (Fig. 1A). Trials involving the same class recorded from different runs (i.e., LR, FR, LF, LX, FX, and XR) were pooled (e.g., for “L” class “L” trials were pooled from LR, LF, and LX runs) forming a re-structured dataset. Thus, in the re-structured dataset four different classes (L, F, R, and X) were derived. The number of trials per class for a single session varied between 45 and 90.

The three ‘task vs. task’ classifiers (LR, FR, LF) were calibrated using the corresponding trials stored in the re-structured dataset. However, in runs when the NeuroSensi game was controlled with a ‘task vs. relax’ (TX) task, i.e., in runs where the character was controlled with LX, FX, or XR task pairs, the same TX decoder was used. The TX decoder was calibrated using T vs. X trials from the re-structured dataset where T trials comprised the L, F, and R pooled trials. To improve the cross-session stability of the calibrated BCI, the final dataset for BCI calibration was prepared by pooling re-structured datasets from multiple sessions acquired prior to the calibration.

#### Triad game for monitoring details of the multi-class classification

The Triad game (Fig. 1C, Additional file 4: Video S4, introduced in 2020) provides real-time continuous visual feedback from each of the four 2-class classifiers. The analogue output of the three ‘task vs. task’ classifiers (LR, FR, LF) are presented using a light blue ball on the three edges of a triangle. Furthermore, the linear combination of the LR, FR, and LF classifier output is presented with an additional coloured ball indicating the composite output of these three ‘task vs. task’ classifiers. The colour of the composite output indicator ball is assigned via the analogue output of the ‘task vs. relax’ (TX) classifier. The colour of the ball indicates whether the command is decoded as the task (green) or relaxed (dark blue) condition. The Triad game provides an opportunity for online monitoring of a combination of the three ‘task vs. task’ and concurrently the ‘task vs. relax’ classification. For example, in session 17 during 2020 the triad game was regularly used by the researcher as a monitoring tool that displayed real-time analogue output from all 2-class classifiers whilst the pilot played the BrainDriver game (described in the next subsection). Furthermore, in 2020, from session 19 onwards, at the beginning of each session the Triad game was also used by the pilot to practice controlling multiple 2-class classifiers in parallel, as a warm-up exercise before the first BrainDriver race practice in the session. However, as the Cybathlon event did not permit the use of add-ons during the competition, the pilot did not use the Triad game in parallel with the BrainDriver game. In the future, the Triad game could be used for acquiring data for BCI calibration. However, this was not the case in 2019 and 2020, when the Triad game was first introduced to the pilot.

#### BrainDriver game to familiarize the pilot for the race in the Cybathlon BCI event

After the pilot learned to control the BCI using the NeuroSensi (in 2019 and 2020) and Triad games (2020 only), the BrainDriver race was used in both years to practice control of the avatar—a virtual race vehicle (Fig. 1B, Additional file 2: Video S2 and Additional file 3: Video S3). The actual track of the BrainDriver race comprised four different zones. There are zones with left and right curves and straight zones with streetlights turned on or off. To maintain the maximal speed of the vehicle, the pilot must produce the correct race command using the 4-class BCI, e.g., left or right arm motor imagery for left or rights turns, feet imagery for “headlight” and relax for “no-control”. If an incorrect command is presented the vehicle is inhibited which decreases speed and increases race completion times, and moreover, presents obvious negative visual feedback to the pilot which enable learnings and error correction strategy development. The pilot was instructed to relax immediately after issuing a command to allow for ‘no control’, or as an alternative strategy to continue to maintain the motor imagery command. “The online BCI” section describes how the controller limits commands and assists in dealing with variation in control performance by the BCI and pilot.

### Data acquisition

**Fig. 2:**
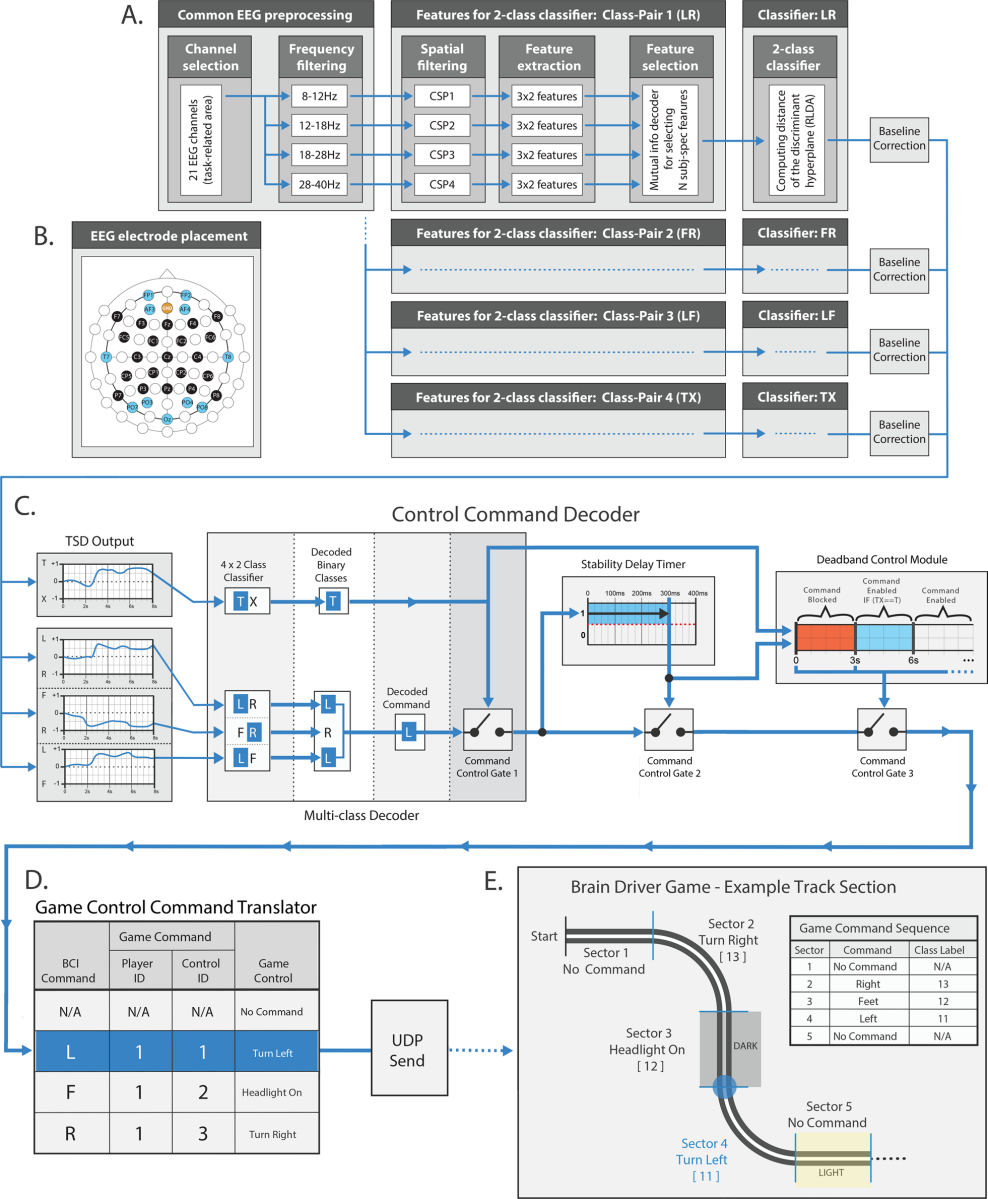
The framework of the BCI; developed for controlling the BrainDriver game. **A** Block diagram of the four 2-class classification modules. **B** EEG sensor placement. The 32 EEG channels are indicated with blue and black from which the 21 blue channels were used to calibrate the control command decoding framework. The ground electrode is indicated with orange. **C** Control command decoder module. **D** Game control command translator module. **E** An example of a track section in the BrainDriver game

The EEG was recorded from 32 EEG channels (Fig. 2B) using a g.Nautilus Research active electrode wireless EEG system (g.Scarabeo) [22] with the EEG reference electrode positioned on the left earlobe. The EEG was high-pass filtered (> 0.1 Hz), notch filtered (48–52 Hz), and digitized (A/D resolution: 24 bits, sampling rate: 250 Hz). The ground electrode was positioned over the AFz electrode location according to the international 10/20 EEG standard (Fig. 2). Communication between the real-time BCI decoder module deployed in Simulink [23] (used for EEG data acquisition and online signal processing) and each of the three games (NeuroSensi, BrainDriver, and Triad) was via the ‘user datagram protocol’ (UDP).

### Calibration of the two-class classification modules

The BCI framework included a filter-bank common spatial patterns (FBCSP) [24] and mutual information (MI) based feature selection [25], a well-established framework used in BCI applications that enable discrimination between imagined movements [26] performed with the left hand (L), feet (F), right hand (R), and relax (X) conditions [27]. The FBCSP-MI module, the core of the online BCI framework (Fig. 2A), was calibrated offline as described below.

#### EEG signal processing

The acquired EEG dataset (“Data acquisition” section) was band-pass filtered in four non-overlapped standard EEG bands (8–12 Hz (mu), 12–18 Hz (low beta), 18–28 Hz (high beta), and 28–40 Hz (low gamma)) using high-pass and low-pass finite impulse response (FIR) filter modules (band-pass attenuation 0 dB, band-stop attenuation 60 dB). The band-pass filtered EEG was downsampled from 250 to 125 Hz. Trial-relevant time intervals between − 2 s before and 8 s after the onset of the 2 s pause [i.e., − 4 s before and 6 s after the onset of task (described in “Experimental paradigms” section)], were epoched out from the filtered EEG dataset for 21 pre-selected EEG channels (indicated with black in Fig. 2B), and stored for spatial filtering. The epoched data using a 1 s to 2 s width classification window enabled comparison of the decoding accuracy (DA) obtained in the 0 to 2 s reference baseline interval (covering the pause period) and during the 2 s to 6 s task interval (after the pause period).

#### Spatial filtering

The common spatial patterns (CSP) method was used to create spatial filters that increase the separability between two classes by maximizing the variance of band-pass filtered EEG signals from one class, while minimizing their variance from the other classes [28]. The linear transformation matrix defined by CSP converts the pre-processed EEG signals into a new vector space defined by the CSP filters.

#### Feature extraction

The number of selected CSP filter pairs for each 2-class classifier for each frequency band was set to three. The time-varying log-variance of the CSP filtered EEG was calculated using a 1 s width sliding window, with a 40 ms time lag between two windows. Thus, the offset (end-point) of the 1 s sliding window was set to cover the time interval between − 1 s before and 8 s after the onset of the pause (covering a 1 s sliding width window, the 2 s pause, and 6 s task intervals).

#### Feature selection

The mutual information (MI) between features and associated target class using a quantized feature space was estimated [25] to identify a subset of features that maximize classification accuracy.

#### Two-class classification

A regularized linear discriminant analysis (RLDA) algorithm from the RCSP toolbox [28] was applied to classify the extracted features. Linear discriminant analysis (LDA) uses a class separator boundary in a linear hyperplane to separate data into two classes. The time-varying analogue output of the classifier, i.e., the time-varying signed distance (TSD), is the time-varying distance between the location of the classifier output and the class separation boundary in the LDA hyperplane. The class assigned to each feature vector depends on the polarity of the classifier output, determined by the relation between the location of the feature vector and the class separator boundary in the hyperplane [29]. The current TSD value is calculated as described in (1):

1 $${TSD}_{n,t}={w}^{F}{x}_{F,n,t}-{a}_{0}$$

where, ${x}_{F,n,t}$ is the features vector at time *t* in the $n$th trial, while ${w}^{F}$ and ${a}_{0}$ are the weights (slope) and bias of the discriminant hyperplane.

In a six-fold cross-validation analysis, the time-varying DA was calculated and compared for each of the four 2-class classifiers using the highest 6, 10, 14, and 18 MI ranked features. The number of highest ranked features that provided a classifier configuration with the highest DA peak in the event-related period of the task (in a 2.4 to 8 s interval of the trial, covering a 0.4 to 6 s interval from the onset of the task) was applied to the online BCI configuration, in the case of each 2-class classifier, separately. We highlight that an optimal number of features were applied to the online BCI which were selected based on which configuration obtained the highest MI rank during the calibration method. Furthermore, as each feature was derived via a CSP weighted linear combination of the band-pass filtered EEG signals, the information content of a feature is not limited to a single EEG channel is comprised of all channels which were applied in the FBCSP framework.

#### Topographical analysis

To identify frequency bands and cortical areas that provide the highest contribution to the peak DA, an analysis was performed using parameters of the calibrated CSP filters and the MI weights for each of the four 2-class classifiers, separately. For the time-varying frequency analysis, the mean values of MI weights were calculated in each analyzed frequency band, and time point, separately. The obtained results were plotted in the form of subject-specific heat maps, indicating the time-varying DA contribution of the frequency bands analyzed. The location of the source activity was plotted using the ‘standardized low resolution brain electromagnetic tomography’ (sLoreta) software package [30] for each 2-class classifier in each frequency band, separately, indicating cortical areas where features provided the highest contribution for calculating maximal DA.

#### Combining trials for different runs and sessions

The objective was to find an online BCI configuration that provides the highest DA with a high level of stability over sessions. Thus, the BCI was calibrated using different datasets that were pooled from different combinations of existing sessions. A cross-session DA analysis was performed for each BCI configuration, wherein the time-varying DA plots were compared using datasets excluded from calibration data. The BCI configuration was selected for the subsequent sessions based on a comparison of the cross-session time-varying DA plots, frequency maps, and topographical maps using the various BCI configurations and objectives described above (i.e., long term stability paired with a maximal level of DA).

### The online BCI

The core module of the online BCI involved the same FBCSP MI based 2-class classification framework (Fig. 2A) for NeuroSensi, Triad, and BrainDriver race which are described in “Calibration of the two-class classification modules” section. However, the post-processing module was different for each of the three paradigms/games.

#### NeuroSensi game

The NeuroSensi game (Fig. 1A, Additional file 1: Video S1) uses only one of the four binary classifiers for controlling the character (i.e., LR, FR, LF, or TX). The baseline of the corresponding TSD signal was calibrated manually and set to zero at the beginning of each run using an offset value. The amplitude of the TSD signal, using a scaling factor, was corrected to a value that enabled the controlled character to move over the controllable area during the game. The corresponding TSD, after the baseline correction, was downsampled to 25 Hz and sent by UDP to the NeuroSensi game. The avatar in the NeuroSensi game was controlled continuously using the post-processed analogue TSD control signal. Jitters in TSD signal were smoothed in the Unity game engine using a linear interpolation method based “lerp” function that smooths the transition between two values over time [31].

#### Triad game

As the Triad game (Fig. 1C, Additional file 4: Video S4) was controlled by the TSD output of each of the four 2-class classifiers, the baseline of each of the four TSD signals was corrected, separately, as described above for the NeuroSensi game. In addition, a smoothing filter option was applied to the baseline corrected TSD calculating the moving average within a 1 s window. The post-processed TSD was downsampled to 25 Hz and sent by UDP to the Triad game. Similarly to the NeuroSensi game, the continuous movement of the game avatars in the Triad game (i.e., one ball on each edge of the triangle plus one more ball in the middle) was smoothed by the Unity game engine using the “lerp” function [31].

#### Cybathlon race: BrainDriver

The avatar in the BrainDiver race (Fig. 1B, Additional file 2: Video S2 and Additional file 3: Video S3) was controlled by the online BCI framework presented in Fig. 2. The online BCI, in addition to the FBCSP-MI and TSD baseline correction modules (discussed above and presented in Fig. 2A), involves a control command decoder module composed of a multi-class decoder, a stability delay timer, a dead-band control module (Fig. 2C) and game control command translator module (Fig. 2D) followed by a UDP unit for sending commands to the BrainDriver platform (Fig. 2E).

The output of the multi-class decoder relies on the baseline-corrected outputs of the four binary (LR, FR, LF, and TX) classifiers. If the output polarity of two of the three ‘task vs. task’ classifiers (LR, FR, LF) are not conflicting and the TX classifier is indicating a task condition (“T”) (i.e., the pilot is not relaxed), the label of the decoded task is forwarded to the next module for a stability check. For example, in Fig. 2C both LR and LF classifiers output indicate the same (“L”) result and the TX classifier indicates that there is an ongoing task (“T”). Therefore, in this example, the decoded (“L”) command passes through on Command Control Gate 1.

To filter out transient responses, the decoded (“L”) command passes through Command Control Gate 2 only if the decoded (“L”) command is maintained in the same condition for a predefined (300 ms) period. If this stability check is matched, the decoded (“L”) command is translated with the game control command translator module to the game control command as shown in Fig. 2D. Finally, the game control command is sent by UDP to the BrainDriver game. An example of a track section and corresponding control commands are illustrated in Fig. 2E (details of the BrainDriver game in “Experimental paradigms” section).

To provide the opportunity for the pilot to reach a relaxed condition before the next command is decoded and to ensure sudden changes in classifier conditions do not interrupt a correct command issued to the vehicle, a dead-band system is activated. The dead-band control module involves a dead-band (DB) timer and a dead-band-break (DBB) timer module. Once a command is sent to the BrainDriver game, the DB timer countdown is activated, which blocks any new commands during the DB period. However, the DBB timer allows the pilot to correct an incorrect command by breaking the dead-band if the TX classifier after a (e.g., 3 s) DBB period detects that a command is being issued for a sufficiently long (e.g., 1 s) period whilst the dead-band is active.

For example, if the length of race zone is approximately 6–8 s and if the intended command is issued just as the avatar reaches a zone, the dead-band will ensure the avatar maintains the associated control for that entire zone, thus maximizing speed. However, if an unintended command is issued at the start of a zone the pilot can attempt to correct it by attempting to issue and produce another command for more than the predefined (e.g., 1 s) period. In such a case a portion of the speedup from that zone may be gained. The dual scenario here improves stability of control and makes the assumption that good commands are more frequent.

To find an optimal configuration that supports the pilot’s control ability maximally, the actual value of the dead-band and dead-band-break parameters were adjusted manually over sessions and runs during the training period. The dead-band was selected in a range between 2 and 8 s, and the dead-band-break timer was selected in a range between 2 and 4 s.

### Statistical analysis

To evaluate differences in race completion times achieved during different periods of the long-term BCI training, paired-sample t-tests were performed on race times achieved in ten races for each period compared (see “Results” section).

To assess the potential causes of fluctuation in performance throughout training and during race days, logarithmic magnitude of power spectral density (PSD) located bilaterally over sensorimotor areas (C3, and C4) and centrally (Cz) were evaluated on different groups of races/sessions where race times differed. As the EEG dataset on the race day in 2020 was not stored due to an oversight, this analysis was performed for the dataset acquired in 2019 only. A three-way analysis of variance (ANOVA) was used to assess differences in 2019 using the following factors and levels: (1) session group (level 1: races from sessions 15 and 16, level 2: from 17 and 18, and level 3: from 19 and 20); (2) electrode site (level 1: C3, level 2: Cz, and level 3: C4); and (3) frequency (levels 1 to 15: frequencies ranging from 12 to 40 Hz in steps of 2 Hz (i.e., level 1: 12 Hz, level 2: 14 Hz, …, level 15: 40 Hz). The ANOVA was two-tailed, with a 95% confidence interval, and was conducted using the Statistical Package for Social Sciences (IBM SPSS Statistics 27.0). A Greenhouse–Geisser correction was applied when sphericity was violated and a Bonferroni correction was applied to pairwise comparisons to control for multiple comparisons.

## Results

Using datasets acquired in 2019 and 2020, an offline analysis was performed to determine which modifications of the current BCI framework and calibration methods improved the pilot’s BCI control accuracy.

### Calibration details

To improve the cross-session stability of the online BCI, calibration was performed using a dataset acquired from multiple sessions prior to the calibration date. To find an optimal combination of sessions to include in BCI calibration dataset, the BCI was calibrated and tested multiple times using data acquired from a combination of different sessions. A dataset involving two to four sessions which provided the highest cross-session DA over sessions near to the calibration date, were used for the BCI re-calibration. Table 1 shows: (1) which sessions were used in the cross-session stability test for selection; (2) sessions which provided the best cross-session accuracy and, therefore, were used for re-calibrating the BCI; and (3) sessions where the re-calibrated BCI was used for game control.

**Table 1:** The list of sessions that were used: (1) based on the cross-session stability test; (2) in BCI re-calibration; along with sessions; (3) where the re-calibrated BCI was used

Sessions used in cross-session stability test to find which sessions provide the most stable BCI configuration	Sessions that were selected in the cross-session stability test and were used in BCI re-calibration	Sessions in which the re-calibrated BCI configuration was used
N/A	N/A	2019 Sessions 1–3 (offline)
2019 Sessions 1–3	2019 Sessions 1–2	2019 Session 4
2019 Sessions 1–4	2019 Sessions 1–4	2019 Session 5
2019 Sessions 1–5	2019 Sessions 2, 4, 5	2019 Sessions 6–20
2019 Sessions 1–5	2019 Sessions 2, 4, 5	2020 Sessions 1–4
2020 Sessions 1–4	2020 Sessions 3, 4	2020 Sessions 5–9
2020 Sessions 1–9	2020 Sessions 3, 4, 5, 6	2020 Sessions 10–22
2020 Sessions 1–22	2020 Sessions 5, 6, 20, 21	2020 Sessions 23–25

### Time-varying decoding accuracy and TSD analysis for 2-class BCI

**Fig. 3:**
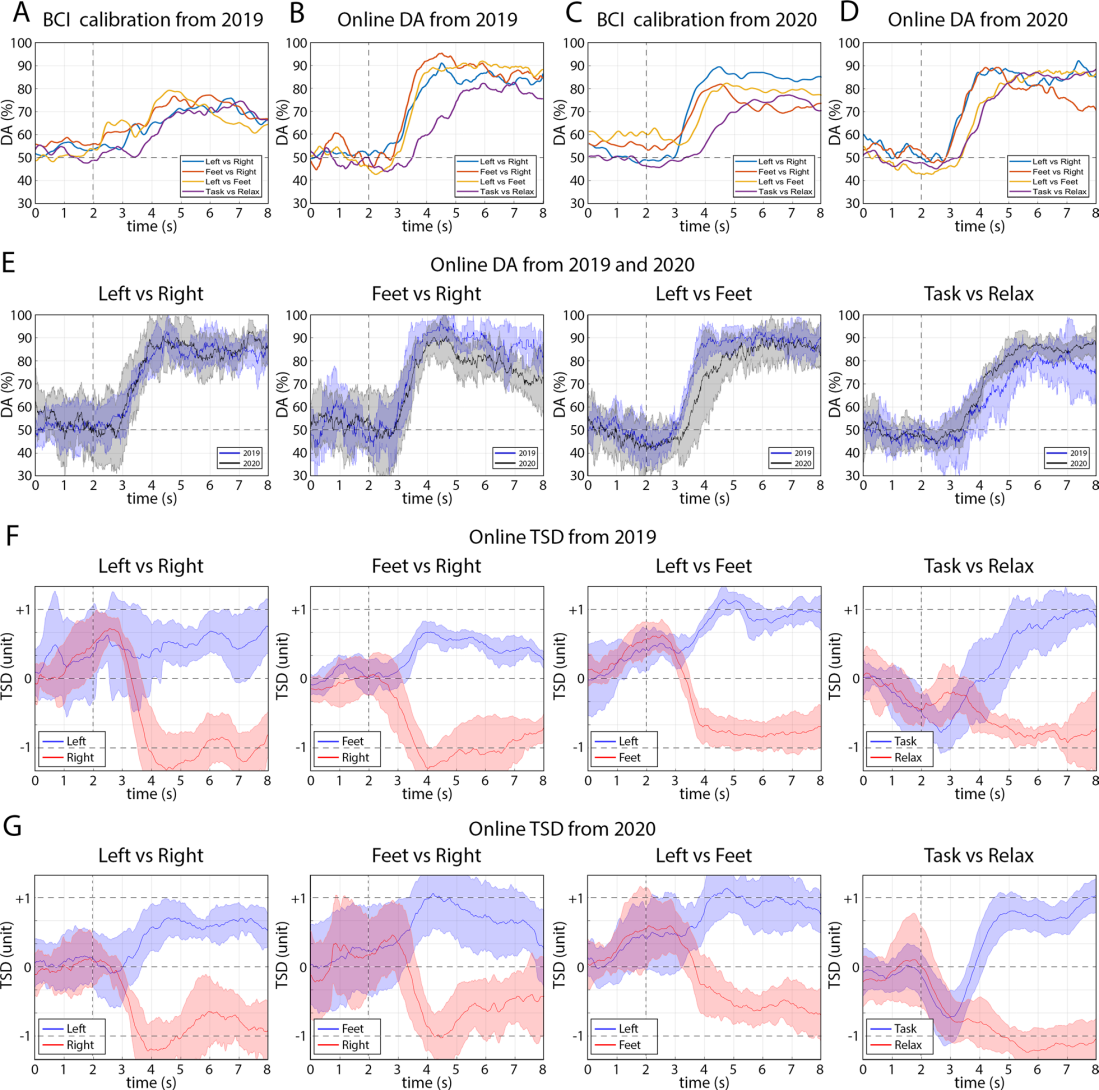
Time-varying decoding accuracy (DA) and TSD results for the 2-class classifiers. **A**–**D** Comparison of the time-varying DA obtained for the four binary classifiers (LR, FR, LF, and TX). **A** Time-varying DA obtained from cross-validation (CV) during BCI calibration using dataset recorded in 2019 at sessions 2, 4, 5. **B** Cross-session average of time-varying DA obtained in 2019 at sessions 6–10. **C** Time-varying DA obtained from cross-validation (CV) during BCI calibration using dataset recorded in 2020 at sessions 3, 4, 5, 6. **D** Cross-session average of time-varying DA obtained in 2020 sessions 7–13. **E** Comparison of time-varying decoding accuracy obtained in 2019 vs. 2020 for each classifier (LR, FR, LF, and TX), separately. **F** and **G** Time-varying TSD values obtained for each binary classifier in 2019 during sessions 6–10 (**B**) and 2020 during sessions 7–13 (**E**). The onset of the task performance is indicated in each plot with a vertical dotted line (at 2 s). The solid lines in shaded areas of **E–G** panels indicate the mean value from analyzed sessions, and the shaded area is the standard deviation

To evaluate how to change the class-specific output of the 2-class classifiers over a trial, the TSD was calculated for each class and plotted along with the time-varying DA for each 2-class classifier (LR, FR, LF, TX), separately, using the dataset acquired in 2019 and 2020. The time-varying DA and TSD outputs of the four binary classifiers are presented in Fig. 3.

Figure 3A and C provide a comparison of time-varying DA obtained in cross-validation performed during calibration of the FBCSP framework applied to the BCI in 2019 and 2020, respectively. A comparison of the time-varying DA obtained for different binary classifiers (LR, FR, LF, and TX) during the online sessions using NeuroSensi is presented in Fig. 3B and D, respectively. Furthermore, a comparison of the time-varying DA obtained from the same binary classifier in 2019 vs. 2020 years is presented in Fig. 3E. The time-varying DA graphs show that DA during the 0–2 s pause period is approximately at chance level (50 ± 10% (mean ± STD)). After the onset of the motor imagery task (the dotted vertical line at 2 s), the online DA reached 90 ± 10% (mean ± STD) (Fig. 3E) and was maintained by most classifiers for a period which was denoted between 4 and 8 s (i.e., from 2 s after the onset of the task until the end of the task period). A comparison of the TSD values obtained for the two classes is presented in Fig. 3F and G using the dataset recorded in online sessions in 2019 and 2020, respectively. The graphs show that during the pause period (i.e., before the onset of the task) the TSD varied in the same range for both classes around the zero baselines and that the TSD separability for the two classes is maximal around 4 s (i.e., 2 s after the onset of the task).

### Frequency analysis and topographical results

**Fig. 4:**
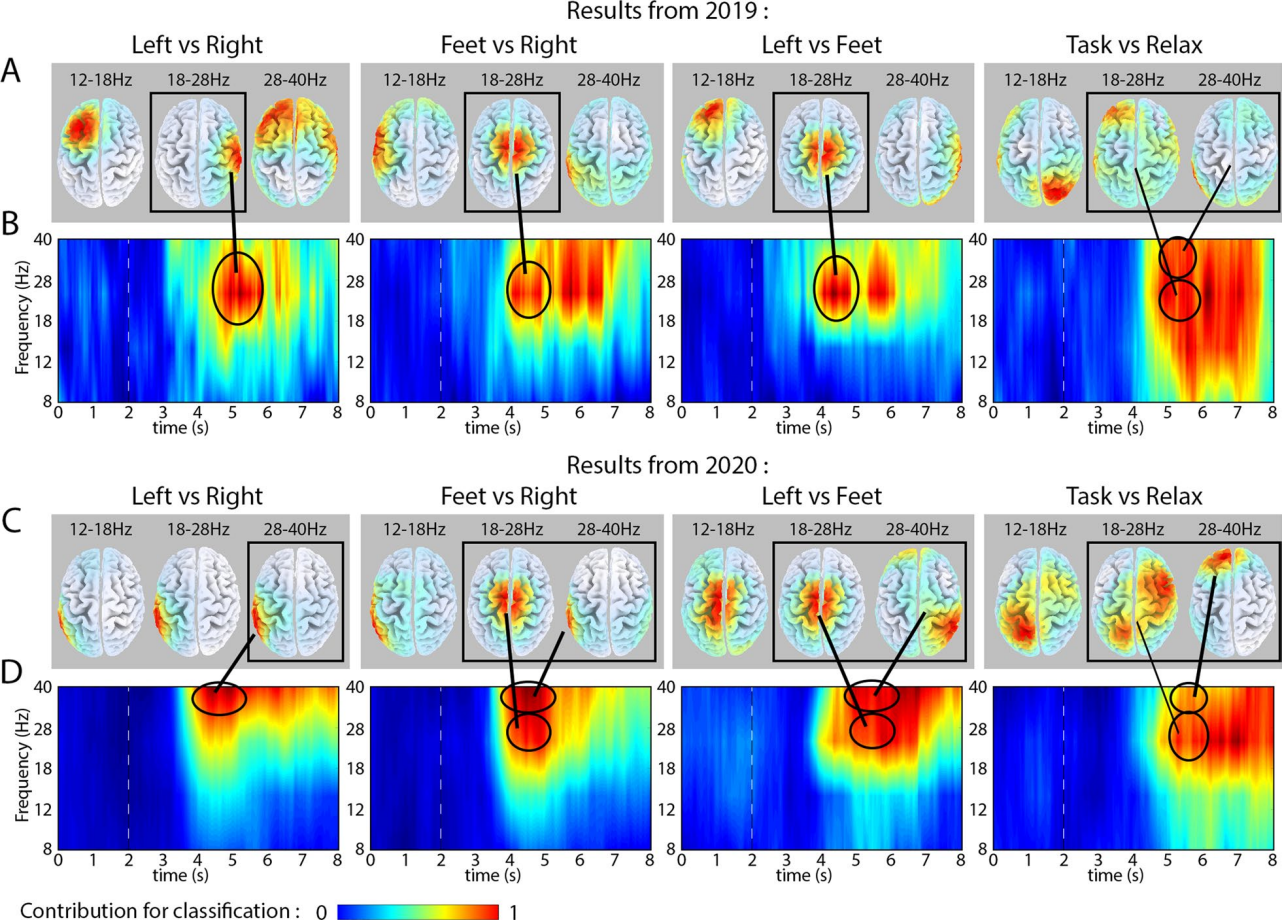
Results of the topographical and frequency analysis. **A**, **B** Results from 2019. **C**, **D** Results from 2020. **A** and **C** Topographical maps of cortical areas providing the highest level of contribution for 2-class classification are indicated in red, separately, for LR, FR, LF, and TX classifiers in the three most prominent (12–18 Hz, 18–28 Hz, 28–40 Hz) frequency bands. **B** and **D** Time-varying frequency maps indicating frequency range providing the highest level of contribution for 2-class classification are indicated in red, separately, for LR, FR, LF, and TX classifiers. The rectangles in the **A** and **C** panels highlight topographical maps that belong to the most prominent frequency range (indicated with ellipses in the **B** and **D** panels)

A comparison of frequency bands and cortical areas providing the highest DA contribution in the four binary classifiers (LR, FR, LF, TX) involved in the BCI configuration applied in the Cybathlon race in 2019 and 2020 is presented in Fig. 4.

The results of the frequency analysis using CSP and MI weights of the BCI that was applied in 2019 indicates an increased level of motor imagery task-related brain activity for each of the three ‘task vs. task’ classifiers (LR, FR, LF) in the 18–28 Hz (high beta) band (Fig. 4B). The highest contribution for the ‘task vs. relax’ (TX) classification, similar to the LF, FR, and LF classification, was obtained in the high beta band. However, regarding the separation of the task and relaxation conditions, in addition to the 18–28 Hz (high beta), the 12–18 Hz (low beta) and 28–40 Hz (low gamma) bands also contribute to high accuracy.

The topographical analysis of the BCI calibrated in 2019 (Fig. 4A) indicated the highest contribution to the four binary classifications was as follows: (1) LR classification, from the high beta oscillations in the right hemisphere of the somatosensory cortex; (2–3) LF and FR classifications, from the high beta oscillations in the central area of the primary motor and somatosensory cortex; (4) TX classification, from the high beta oscillations in the left hemisphere of the prefrontal cortex, coupled with the low gamma oscillations in the left hemisphere of the occipito-temporal cortex. Furthermore, for the TX classification, the low beta oscillations also provided a reasonably high contribution in the right hemisphere of the visual sensation associated area in the parietal occipital cortex.

In 2020, the results of the frequency (Fig. 4D) and topographical (Fig. 4C) analyses indicated differences in the frequency and topographical maps compared to those obtained in 2019. In 2020, the highest contribution to the four binary classification is obtained in cortical areas as follows: (1) LR classification, from the low gamma oscillations in the left hemisphere of the somatosensory association and occipito-temporal cortex; (2) FR classification, from the high beta oscillations in the central area of the primary motor and somatosensory cortex, coupled with the low gamma oscillations in the left hemisphere of the somatosensory association and occipito-temporal cortex; (3) LF classification, from the high beta oscillations in the central area of the primary motor and somatosensory cortex, coupled with the low gamma oscillations in the right hemisphere of the somatosensory association and occipito-temporal cortex; (4) TX classification, from the high beta oscillations in the left hemisphere of the somatosensory association cortex, the right hemisphere of the premotor, and primary motor cortex, coupled with the high beta oscillations in the left hemisphere of the prefrontal cortex.

### BrainDriver race scores, baseline correction, and dead-band configuration

**Fig. 5:**
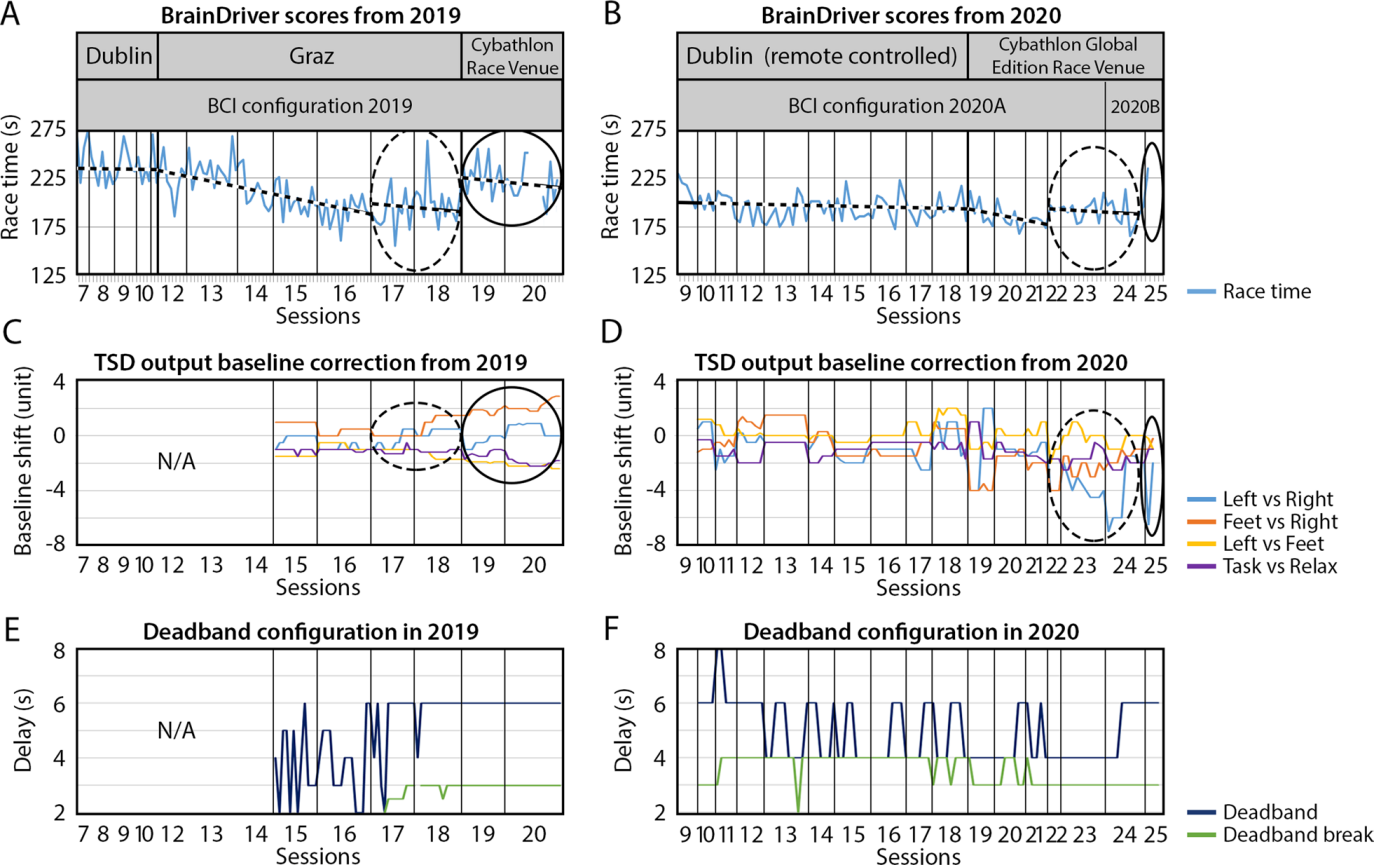
The BrainDriver race times were achieved by the pilot in 2019 and 2020 and changes in key parameters throughout the training period. **A** and **B** BrainDriver race competition time achieved in 2019 and 2020, respectively. **C** and **D** TSD outputs baseline correction applied to the 2-class classifiers of the online BCI in 2019 and 2020, respectively. **E** and **F** Dead-band and dead-band-break configuration applied to the online BCI in 2019 and 2020, respectively. Minor ticks at horizontal axes of graphs presented in (**A**–**F**) panels indicate values obtained, separately, in each run of the corresponding session. Dashed (and solid) oval highlights sessions near (and during) the competition day when the pilot’s BCI control ability decreased (i.e., race completion time increased) compared to earlier sessions

Each year, after undertaking practice sessions using the NeuroSensi game, our pilot’s training focused on the Cyathlon race paradigm: BrainDriver. The race completion times achieved by the pilot during training and race day performances in 2019 and 2020 are presented in Fig. 5.

#### BrainDriver race times

In both years, during the training period, the race completion time decreased over sessions. However, around four days before the competition, each year, the required time for finishing the BrainDriver race increased (highlighted with the dotted oval in Fig. 5A and B). Furthermore, on the day(s) of the competition, the required time to finish the BrainDriver race increased, resulting in substandard performance in the final competition race compared to earlier training performance (e.g., up to session 16 in 2019 and up to session 21 in 2020).

A statistical analysis indicated significant improvement in race completion times (paired-sample t-test, *p* < .001) between the first ten races in 2019 (from the beginning of 2019 session 7) and in the last ten race attempts during training before the degradation in the pilot’s performance before the competition (at the end of 2019 session 16). No differences were observed in 2019 race times (paired-sample t-test, *p* > .05) in ten races before the end of the training phase (closed at the end of 2019 session 16) and after the beginning of the pre-competition period (counted from the beginning of 2019 session 17) even though during the latter which was closer to the competition day the race time varied significantly more. Furthermore, the ten race completion times in 2019 (counted from the beginning of 2019 session 19 and into competition days) were significantly higher than those during the ten races after the beginning of 2019 session 17 (paired-sample t-test, *p* < .002).

A similar analysis was performed using race times obtained in 2020. Completion times in the first ten races attempt (recorded after the beginning of 2020 session 9) were significantly higher (paired-sample t-test, *p* < .001) compared to that achieved in ten runs before the time when the pilot’s performance started to decrease (i.e., before the end of 2020 session 21). Moreover, the game completion times increased significantly in ten race attempts at the beginning of the pre-competition period (counted after the beginning of 2020 session 22) compared to the last ten race attempts performed at the end of the training phase (before the end of 2020 session 21) (paired-sample t-test, *p* < .009).

Finally, an analysis was performed evaluating possible transfer of the learned BCI control skills between 2019 and 2020. The race times obtained in the last ten gameplays before the improvement in the pilot’s performance stopped (at the end of 2019 session 16) were compared to race times obtained in the first ten races in 2020 (from the beginning of 2020 session 9). No significant difference between the two compared periods (paired-sample t-test, *p* > .05) was observed even though there was approximately 1 year of no training between these two periods.

**Fig. 6:**
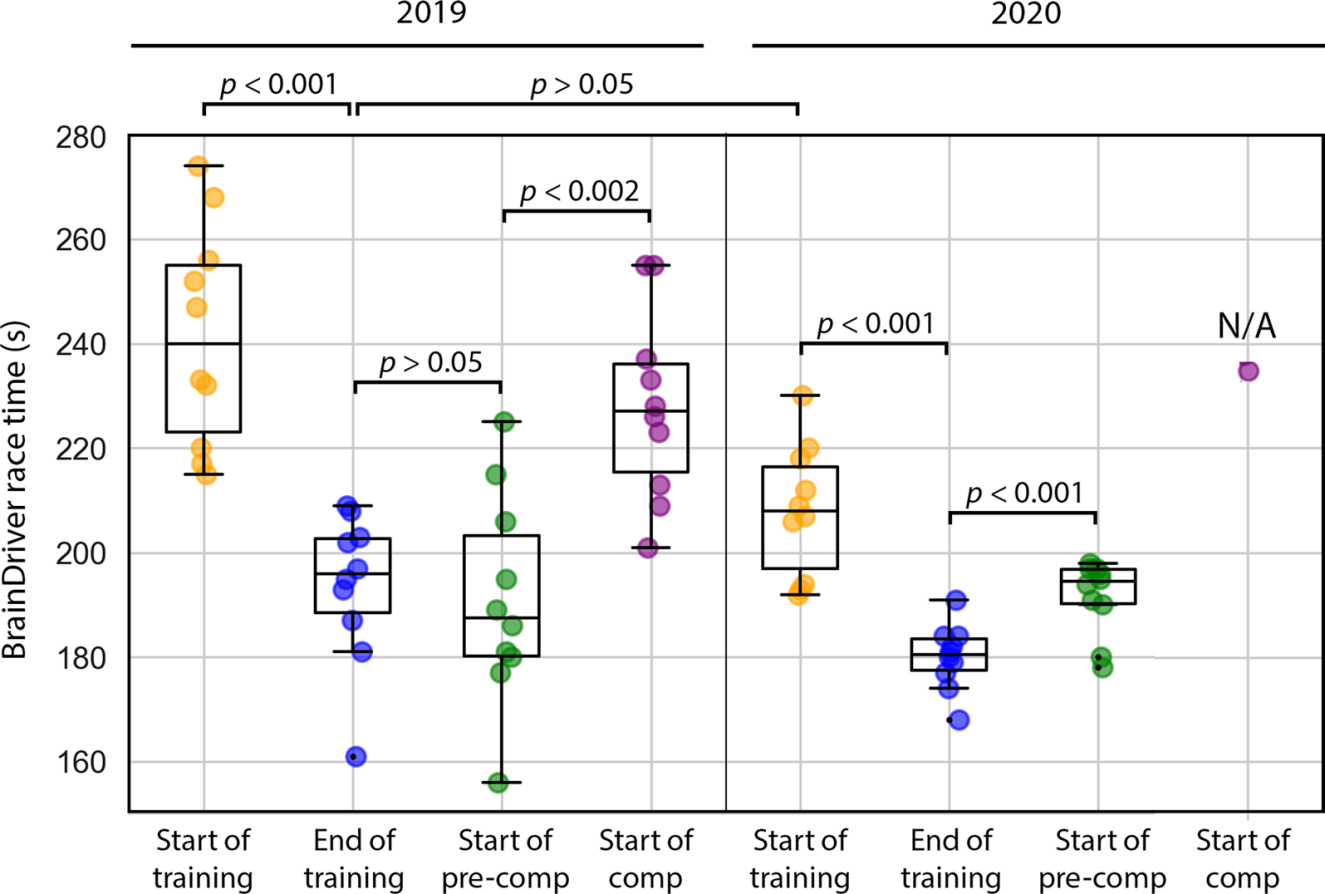
Comparison of BrainDriver race times obtained in different periods of the BCI training. Coloured dots displayed in the boxplots indicate race time values obtained from ten BrainDriver races during the period indicated in the horizontal axis (i.e., in ten runs after the beginning of the training phase, ten runs before the end of the training phase, ten runs after the beginning of the pre-competition phase, and ten runs after the beginning of the competition phase, for both 2019 and 2020, years). The box extends from the lower to upper quartile values, with a line at the median. The whiskers extend from the box to show the range of displayed race time values. *p* values obtained from paired-sample t-tests are also presented

Figure 6 compares BrainDriver race times obtained in different periods of the BCI training.

#### Baseline shift and dead-band control parameters

The baseline shift on the TSD of the 2-class classifiers (Fig. 5C and D) is correlated with the increase in race times (Fig. 5A and B) as highlighted with a dotted and solid oval.

To find a BCI configuration that maximally supports the pilot’s control ability, the values of the dead-band and dead-band-brake timers were set over sessions manually. The applied values of the dead-band and dead-band-brake parameters are presented in Fig. 5E and F. Based on manual observation of the game completion times and feedback information reported by the pilot about which configuration best supported control ability, the BCI was configured for the final challenge in both years, 2019 and 2020, using a 6 s dead-band and a 3 s dead-band-break time.

### BrainDriver race performance and EEG power spectral density (PSD)

ANOVA was performed to evaluate a possible connection between the change in BrainDriver race times and the power spectral density (PSD) of EEG during BrainDriver race control, across the 2019 sessions and runs (“Statistical analysis” section). As the EEG dataset on the race day in 2020 was not saved due to an oversight, this analysis was performed for the dataset acquired in 2019, only.

**Fig. 7:**
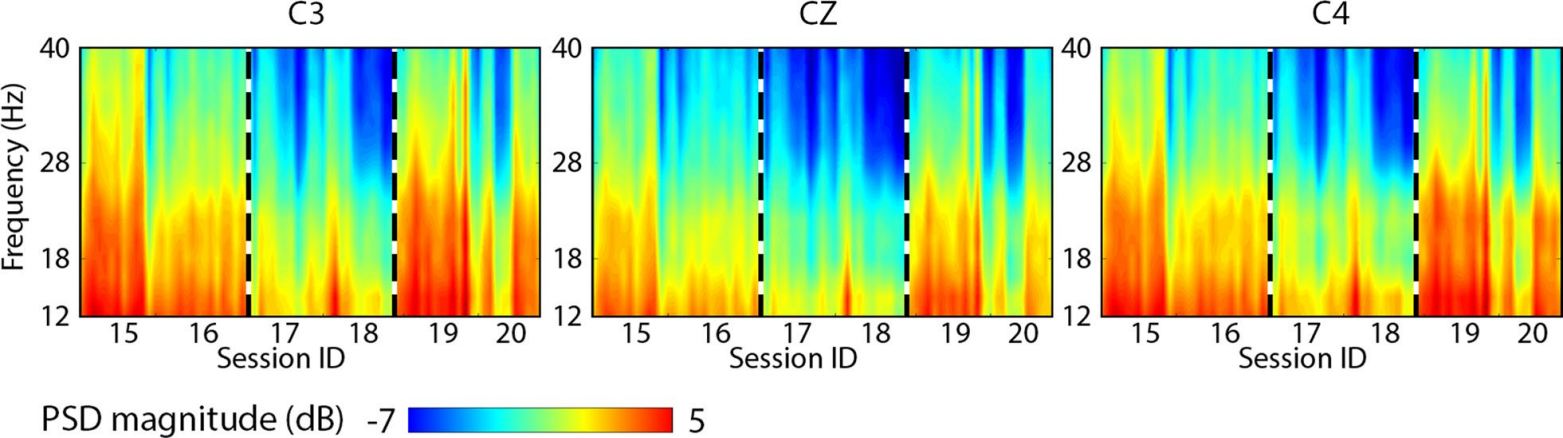
Power spectral density (PSD) maps obtained from BrainDriver game EEG records in 2019 sessions 15–20. The PSD maps show the logarithmic magnitude for three EEG channels (C3, CZ, C4) that provided a prominent contribution to the control of the BrainDriver in 75 individual games during sessions 15–20 (results from individual games corresponding to the indicated sessions are presented in the horizontal axis). Vertical dotted lines separate session groups 15–16 from 17–18 and 19–20

Figure 7 shows the averaged logarithmic PSD magnitude calculated for each analyzed run, in a 12–40 Hz frequency range, for session groups 15–16, 17–18, and 19–20 at C3, CZ, C4 electrodes which are located centrally and bilaterally over sensorimotor areas. Main effects were significant for the session group, electrode site and frequency (*F*_*(2, 46)*_ = 21.65, *p* < .001, $\upeta _{{\text{p}}}^{2}$ = .49, *F*_*(1.2, 27.49)*_ = 2604.79, *p* < .001, $\upeta _{{\text{p}}}^{2}$ = .99, and *F*_*(3.04,69.87)*_ = 2592.69, *p* < .001, $\upeta _{{\text{p}}}^{2}$ = .99, respectively). Significant interaction effects were found for session and electrode (*F*_*(1.96, 45.19)*_ = 8.19, *p* = .001, $\upeta _{{\text{p}}}^{2}$ = .26), session and frequency (*F*_*(28, 644)*_ = 9.67, *p* < .001, $\upeta _{{\text{p}}}^{2}$ = .3), electrode and frequency (*F*_*(28, 644)*_ = 166.13, *p* < .001, $\upeta _{{\text{p}}}^{2}$ = .88), and a three way interaction between session, electrode and frequency was also found to be significant (*F*_*(56, 1288)*_ = 7.74, *p* < .001, $\upeta _{{\text{p}}}^{2}$ = .25). Pairwise analyses, which were corrected for multiple comparison effects using a Bonferroni correction, revealed that the main effect for session was due to a significant difference between session 2 and session 1 (*MD* = 1.9, *SE* = .31, *p* < .001), and session 2 and session 3 (*MD* = 1.69, *SE* = .34, *p* < .001) only, while the main effect for electrode was due to significant differences between all three electrodes (C3 and Cz—*MD* = 1.1, *SE* = .021, *p* < .001; C3 and C4—*MD* = .168, *SE* = .008, *p* < .001; C4 and Cz—*MD* = .92, *SE* = .017, *p* < .001). Finally, the main effect for frequency was due to significant differences between all frequencies (*p* < .001) except for between 18 and 20 Hz (*p* = .18). The interaction between session and electrode was driven by a significant difference at all three electrode locations during session 1 compared to session 2 (C3—*MD* = 2.03, *SE* = .33, *p* < .001; Cz—*MD* = 1.83, *SE* = .29, *p* < .001; C4—*MD* = 1.86, *SE* = .31, *p* < .001) and also during session 3 compared to session 2 (C3—*MD* = 1.77, *SE* = .37, *p* < .001; Cz—*MD* = 1.64, *SE* = .32, *p* < .001; C4—*MD* = 1.65, *SE* = .34, *p* < .001). The session by frequency interaction occurred due to significant differences between session 1 and 2, and between session 2 and 3 (but again, not between session 1 and 3, across all fifteen frequencies (*p* < .001), while the interaction between electrode and frequency was due to significant differences between each frequency at all three electrode sites (*p* < .001). The three-way interaction was found to be due to significant differences in the PSD for all frequencies, as measured at C3, Cz, and C4 electrode locations, during session group 1 and session group 3, when compared to session group 2 (*p* < .01).

### BrainDriver race times and frequency bands providing the highest DA contribution

During pre-competition sessions, performed before competition races, a decrease occurred in the pilot’s BrainDriver game control ability (Fig. 5A and B). Based on the NeuroSensi dataset acquired in 2020, an analysis was performed to investigate a possible connection between the decrease in BCI control ability and the change in frequency bands providing the highest contribution to 2-class classification, comparing results from the final days of the BCI training period (sessions 19–21) and pre-competition days (sessions 22–24). For the analysis, the classifiers were trained separately for the two options, i.e., when the training dataset was pooled from sessions 19–21, and sessions 22–24.

**Fig. 8:**
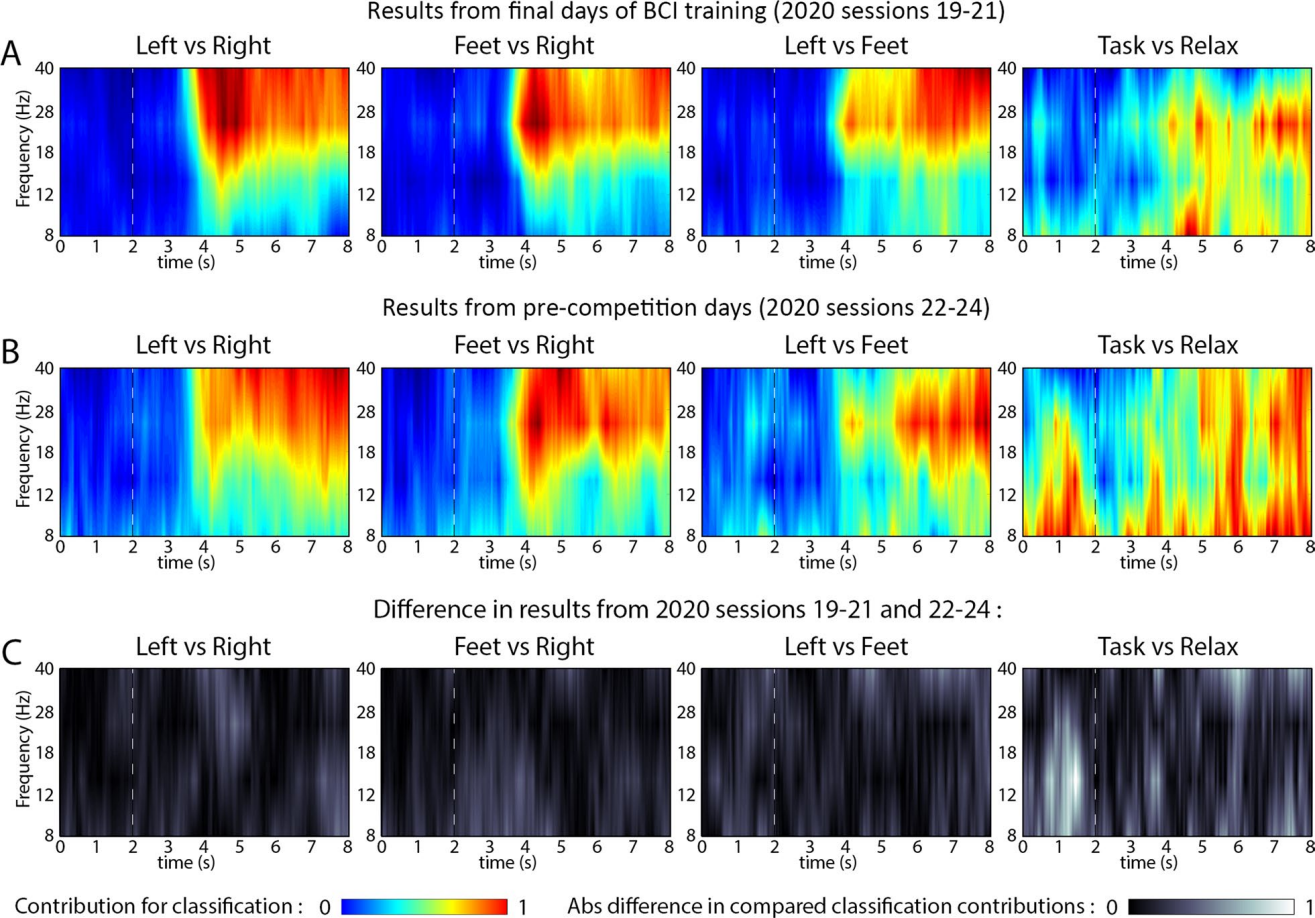
Comparison: DA contribution of frequency bands obtained in 2020 for final days of the BCI training period (sessions 19–21) vs. pre-competition days (sessions 22–24). Time-varying frequency maps indicating frequency range providing the highest level of contribution for 2-class classification using datasets recorded in 2020 for final days of the BCI training period (sessions 19–21) (**A**) and for the pre-competition days (sessions 22–24) (**B**). The absolute value of the difference in (**A**) and (**B**) frequency maps is indicated in (**C**)

The results of the analysis (Fig. 8) for each ‘task vs. task’ classifiers (LR, FR, LF) shows that the task-related DA contribution of the 18–28 Hz (high beta) and 28–40 Hz (low gamma) bands show a relatively high value (red area) for the final days of the BCI training period in sessions 19–21 (Fig. 8A) closer to the onset of the task (dotted vertical line) compared to results obtained for pre-competition days in sessions 22–24 (Fig. 8B). As indicated by the lighter coloured areas in Fig. 8C, the highest difference in DA contribution for ‘task vs. task’ 2-class classification between the two session groups was obtained during the task period around 4–5 s (i.e., 2–3 s after the onset of the motor imagery task).

In the case of the ‘task vs. relax’ (TX) classification, the DA contribution map obtained from the two-session groups showed more diverse non-focal patterns, especially in the 8–12 Hz (mu) band and signifiant difference in the baseline period between the session groups in the BCI training period (sessions 19–21) and pre-competition days (sessions 22–24). Out of all the classifiers the TX appears the least stable between the session groups and without clear distinction between baseline and event related periods in 2020.

## Discussion

This paper provides an overview of a long-term pilot training and BCI strategy implemented in preparation for the Cybathlon BCI race event in 2019 and 2020. In both years, the initial phase of the project focused on calibrating the 2-class classifiers using the NeuroSensi game. Our results show that decoding accuracy increased during the online sessions following the calibration period. This observation is in line with similar studies that have demonstrated improvements in the Cybathlon pilot’s BCI control ability due to long-term practice periods—although this has not always translated into high performance on the day of the event, as was the case with the current study and for both Benaroch et al. [9] and Turi et al. [17]. Possible reasons for this drop in race-day performance are discussed along with suggested strategies to address the issue.

### Analogue TSD output of the 2-class classifiers

The TSD results from the current study indicate that although the BCI was re-calibrated between 2019 and 2020, the timing of the pilot’s accuracy control strategy remained consistent during this time. The TSD output of the ‘left vs. right’ (LR) classifier between 0 and 3 s (reference baseline-related interval) in 2020, shows less fluctuation in positive or negative directions compared to that obtained in 2019. The more balanced TSD track deviation, before the effect of the class-specific motor imagery task in 2020, may be because the pilot was asked to maintain a relaxed mental state whilst keeping the controlled character in the center position (at the zero baseline) during the pause period of the ‘task vs. task’ runs in 2020, but not in 2019. Furthermore, the time course of the TSD values for the ‘task vs. relax’ (TX) classifier within a 2 s width interval around the onset of the task (between 1 and 3 s in the trial) shows that the controlled character moves in the negative direction. The above-described effect is more clearly observable in the 2020 results (Fig. 3G, ‘task vs. relax’) compared to the results from 2019 (Fig. 3F, ‘task vs. relax’), indicating that the pilot followed the instructions and tried to relax during pause periods of the TX runs, especially in 2020.

### Frequency and topographical analysis

The comparison of the frequency and topographic analysis results obtained in 2019 and 2020 using CSP and MI parameters of the calibrated BCI, indicate a similar pattern each year (Fig. 4). However, the patterns obtained based on the 2020 BCI configuration involve some specific features that are not evident in the 2019 BCI. The performance in 2019, for each binary classifier, relied on a single area in the frequency and topographical maps (Fig. 4A and B). However, in 2020, for most binary classifiers, two separate areas provide similarly high contributions for the 2-class classification (Fig. 4C and D). For example, the ‘feet vs. right’ (FR) classification in 2019 relied mostly on 18–28 Hz (high beta) oscillations in the central area of the primary motor and somatosensory cortex—a cortical area commonly activated when able-bodied participants perform the task associated with a kinaesthetically imagined feet movement. This observation is in line with the findings reported by Müller-Putz et al. [32], which reveal a post-movement beta rebound within a mean range of 17.3–29.7 Hz. For the FR classification in 2020, in addition to the high beta activity in the central area of the primary motor and somatosensory cortex, a similarly high contribution was obtained from low gamma oscillations in the left hemisphere of the somatosensory association and occipito-temporal cortex. Thus, the above discussed CSP-MI patterns obtained in 2020 indicate task-specific cortical activity for both compared tasks (‘feet vs. right hand’), as opposed to highlighting only one task (‘feet’), as was the case in 2019.

### Performance of the Ulster University team

Each year, after some initial NeuroSensi game sessions, which served to help the pilot achieve control confidence in the 2-class paradigm, the focus of the sessions turned to the BrainDriver race paradigm. The BrainDriver race completion time during the training period improved significantly. In 2019 completion times ranged from 274 to 156 s over 17 days—including 10 sessions wherein the completion times in the first of these ten sessions (session 9, Fig. 5A) was 255 ± 24 s (mean ± std), reaching 191 ± 14 s in the last of these ten sessions (sessions 16, Fig. 5A).

The game completion time in 2020 ranged from 230 to 168 s over 18 days—including 13 sessions wherein the completion time in the first of these thirteen sessions (session 9, Fig. 5B) was 214 ± 14 s (mean ± std), and reached 181 ± 4 s in the last of these thirteen sessions (session 21, Fig. 5B). However, on both competition occasions, towards the race date, the pilot’s performance in the race decreased significantly. For reference, the winning race times were 183 s and 172 s in 2019 and 2020, respectively.

The results confirm that not only did the multi-session online training with BrainDriver race attempts have a positive impact on the pilot’s performance, manifested in an increased BCI control ability that enabled the pilot to achieve competitive race completion times, but also highlights that experience gained in 2019 was transferred to performance in 2020. This observation resulted in race completion times being achieved at the beginning of the training performed in 2020 which were in the same range as the best race completion times achieved at the end of the training performed in 2019 (paired-sample t-test, *p* > .05).

### Performance of the competing pilots

In terms of other competitors, the NITRO 1 team, Benaroch et al. [9] reported that the game completion time of their pilot fluctuated between 250 and 340 s during seven training sessions before the Cybathlon BCI series event in 2019. However, their pilot could not finish the track within the 4-min limit. The NITRO 2 team, Turi et al. (2021) reported that in the final competition of the 2019 Cybathlon BCI series their pilot completed 390.5 m in the 500 m long virtual track within the 240 s limit [17] but did not note details of the game completion time achieved by their pilot during the training period. For the Mirage 91 team, Hehenberger et al. [33] reported that their pilot’s performance showed a constant improvement over 14 months of training including 26 game-based sessions for the 2019 Cybathlon BCI series and 2020 Cybathlon Global Edition. The BrainDriver race completion time improved from 255 ± 23 s to 225 ± 22 s (mean ± STD). For the SEC FHT team, Robinson et al. [34] also reported improvement in their pilot’s performance over a 9-month training period involving 15 sessions, with BrainDriver competition time varying between 310 and 214 s.

The best three ranked teams completed the track in the final challenge of the Cybathlon BCI series (2019) in the following order. Rank 1st: WHI team (500 m within 183 s), Rank 2nd: Mirage 91 team (500 m within 229 s), Rank 3rd: NeuroCONCISE team (386 m within the 240 s limit) [17, 35]. The best three ranked teams in the 2020 Cybathlon Global Edition completed the track in the following order: Rank 1st: WHI team (500 m within 172 s), Rank 2nd: MAHIDOL BCILAB BCI team (500 m within 176 s), Rank 3rd: Neurorobotics team (500 m within the 213 s). Our team, the Ulster University NeuroCONCISE team, completed the 2020 Cybathlon Global Edition, Rank 6th (452 m within the 240 s limit) [36]. We applied a similar strategy in the 2016 Cybathlon event, with the same pilot, but without a dedicated rest vs. task classifier. While the Cybathlon 2016 data is not comparable due to differences in the race track and total race time, our pilot achieved the 3rd best time of all competitors in the competition, but had a poor qualifying lap which meant being awarded 6th place overall [3].

### Potential factors affecting the NeuroCONCISE pilot’s performance

For a few days before the competition each year, the race completion time achieved by the pilot from session to session decreased, indicating an improvement in BCI control ability and/or a more refined parameters selection for the BCI. However, over the days directly before the competition day each year, a significant increase and or variability in the race completion times occurred (dotted oval area in Fig. 5A and B). Furthermore, on the day of the competition, the race completion time followed this trend (solid oval area in Fig. 5A and B). During the last six sessions before the competition in 2019 when this negative effect was observed, the BCI configuration had not changed, nor was there a change in the game control strategy reported by the pilot. There may be several factors associated with this change in performance, including increased arousal and stress levels, fatigue, and/or changes in living and dietary patterns, e.g., the pilot was living away from home for extended periods during the lead up to race day.

Benaroch et al. [9] demonstrated that their Cybathlon pilot’s strategy of adapting their brain patterns to match the training data distribution helped to improve BCI control. However, they also report that they were not able to translate this to event day performances, which is consistent with our findings. Additionally, Turi et al. [17] reported a similar outcome for competition day results, citing multivariate factors that influence and potentially disrupt pilot performance, including training time, change in routine, differences in the training method with the final game, and differences in training and event environment.

A longitudinal study involving another Cybathlon pilot who has tetraplegia [34] investigated factors affecting the long-term use of their system by analysing several performance indicators including activations maps, completion time, classification, and the personal experience of the pilot, by measuring their subjective experience of both their physical and mental readiness on a scale of 1 to 5. The findings support the use of a closed-loop calibration system with real-time feedback, due to better online median classification performance, compared to open-loop calibration paradigms, and improved pilot engagement. Although only a single subject study, the team recommends striving to keep the training paradigm closely matched with the final event by including closed-loop real-time feedback. This strategy helps to boost the classification performance whilst increasing brain activations due to the increased engagement felt by the pilot.

Promisingly, not all entrants reported a decrease in performance on the event day. Hehenberger et al. [33] described a correlation between external influencing factors and performance on their final race. As was the case for the current study, the authors describe the pilot’s increased performance over time, but did not observe a decrease in performance during the final races. Moreover, the pilot achieved a personal best in his performance at the 2019 Cybathlon, leading the authors to speculate that their pilot performs better in front of an audience.

### Factors of arousal and feedback on BCI performance

In an attempt to understand how to mitigate all negative influences on pilot performance, it has been argued that the peak performance of paralympians is driven not only by psychological factors, but through their convergence with a sufficient support network, lifestyle, and attuned methods of performance [37]. Hence, recent research has focused on counteracting the effects of these variables. One approach has been to use neurofeedback to improve BCI task performance through training self-regulation of arousal states via attention mechanisms [38]. Cognitive control underlies executive function within the brain and interacts critically with arousal systems to activate approach-avoidance behavior [38, 39]. This interaction is the principal behind the Yerkes–Dodson law—a psychological concept that posits that an optimal (moderate) level of arousal is necessary for improved task performance—while a low level of arousal reduces motivation and a high level of arousal negatively impacts cognitive information processing, thus impairing task performance. Therefore, this relationship between the state of arousal and performance on a task is best described as an inverted-U, known as the Yerkes–Dodson curve [38, 40].

Arousal levels have been found to influence sensory-motor cognition—spontaneous high-frequency oscillations known as “pilot-induced oscillation” (PIO) are generated when performing a high-consequence task. These unstable oscillations have deleterious effects on performance when amplified by the pilot’s over-correction of small errors in control [38, 41]. Faller and colleagues (2019) used audio feedback in a closed-loop neurofeedback BCI, comprising a synthetic slow heartbeat (60 bpm), which became louder with increased arousal, to train users to self-regulate their arousal levels while performing a virtual reality (VR)-based boundary avoidance task (BAT). Task performance improved significantly for the users who received veridical feedback compared to those in the sham or no-feedback control conditions. This result was corroborated by heartrate variability (HRV) data and measures of pupil dilation, which indicated a learned ability to shift arousal state and increase task performance through neurofeedback training.

However, it remains uncertain whether the advantage afforded by feedback would be maintained in the absence of that feedback during the competition itself. Mindfulness training could provide a more sustainable approach to the self-regulation of arousal, via underlying cognitive mechanisms. Mindfulness training has been shown not only to have a positive impact on stress reduction [42] but is also gaining momentum in the BCI research community with some studies showing an improvement in performance of almost 20% for those using mindfulness over a control group [43]. Mindfulness is a metacognitive process as it requires self-regulation of attention to control cognitive processes while simultaneously monitoring conscious experience [44]. Individuals who are experienced in meditation skills have been found to demonstrate higher resting SMR power, a more stable resting mu rhythm, and greater BCI control, compared to those who have not practiced meditation techniques [45]. Furthermore, mindfulness-based stress reduction (MBSR) training has been found to improve BCI learning and performance on BCI tasks that communicate the user’s intent via motor imagery commands and volitional rest [46]. The mechanism for this improvement is brought about by the user’s ability to volitionally increase alpha-band neural activity as a consequence of the MBSR training. Stieger et al. [46] evidenced an increase in alpha activity recorded during the user’s volitional resting-state, across MBSR sessions, which was correlated with mindfulness practice and predicted BCI performance. Strategies to reduce challenges and stress when preparing for the competition include anticipation and preparation through detailed planning, including contingency planning, and expectation management, i.e., focusing on the process rather than the outcome [47]. Training in MBSR would fit with these approaches to stress management and enhance BCI task performance under the pressure of competition.

### Distractors and external factors

Factors that impact SMR-BCI performance that are less dependent on the BCI user and more dependent on external elements include distractors, time spent training, various types of feedback, and features of the EEG system and the data preprocessing algorithms [40]. For example, González-Franco et al. [48] whose research studied the influences of positive and negative visual feedback on motor imagery task performance using EEG and electrocardiography (ECG), found that over-biased negative feedback caused mental stress that is detected in the form of significantly higher heart rate variability (HRV), compared to sessions where over-biased positive feedback was presented, and accuracies correlated with the polarity (−/+) of the biased feedback. If a pilot experiences a drop in performance and some negative feedback for any reason during the training sessions immediately preceding the competition day when pressure to perform well and competition anxiety may be heightened the effect of this anxiety may further negatively impact performance and degradation in performance spirals. Race teams engineers may also begin to get anxious when seeing these performance changes and thus start to adjust BCI parameters to counteract and adapt to these changes resulting in the pilot experiencing different feedback than expected and thus further negative effects. This mutual adaptation or perhaps maladaptation can result in significant performance degradation quite quickly. Adaptive feedback, or BCI setup, that limits the negative feedback may be an alternative strategy where the onus is on the BCI to deal with the changes in the pilot’s affective state and certainly further research is required to address this human machine learning dilemma (involving the human pilot, the BCI machine and the human engineer or engineers who are also in the loop).

Performance may be improved by replacing the BCI framework proposed here with an adaptive BCI method that could update the BCI in real-time and adapt to the pilot’s actual mental state [49]. In the lead up to the competition, the average TSD was offset from zero (indicating classifier bias to one of the classes). Even manual correction (offsetting) before each session was not enough to counteract the drift. This baseline drift is a well-known issue in BCI and is associated with changes in the distribution of the features, i.e., covariate shift [50, 51]. As shown in Figs. 7 and 8 we observed changes in the temporal evolution of the frequency response and feature importance changes over time, in addition to the inclusion of some features that negatively impact performance. Although more difficult to manage, an adaptive classifier approach [52] or feature adaptation [53] or data space adaption approach [54, 55], may combat this issue and help maintain performance regardless of the pilot’s affective state.

### Study limitations

The BCI and race strategy proposed here (Fig. 2) requires the task vs. relax’ (TX) classifier to act as a game-control command gating mechanism which oversees when a command is sent to BrainDriver or when a ‘no-control’ state is intended. As the BrainDriver command gating mechanism relies on the time-varying DA of the TX classification, it can have a significant impact on the pilot’s ability to: (1) maintain a stable ‘no-control’ state after a correctly classified game-command; and (2) immediately send a new game-control command to correct a mistake after having sent an incorrect command. We observed that each of the three ‘task vs. task’ classifiers (LR, FR, LF) achieved higher DA and lower DA peak latency compared to the ‘task vs. relax’ (TX) classifier (Fig. 3E). Furthermore, when comparing the TX classifier to other classifiers we also observed that: (1) the DA contribution levels from different frequency bands for the TX classifier were much more diverse; and (2) the difference between contribution patterns obtained for the final training sessions and the competition period were maximal the TX classifier (Fig. 8), signifying least stability. The lower performance of the TX classifier may, therefore, have been influenced more by the pilot’s relax state variability as race day approached and thus overall negatively impacted the performance disproportionally to other classifiers.

Therefore, future work will focus on devising methods to train the pilot on how to use the TX classification more reliably, e.g., by replacing the NeuroSensi game-based multiple 2-class classification training with a Triad game-based training framework, and generally training resting state maintenance and relaxation techniques.

Next, as described in “Calibration of the two-class classification modules” and “Calibration details” sections, a cross-session DA analysis was performed to find a BCI configuration that resulted in the most stable and highest DA peak performance obtained over multiple test sessions. The datasets, which were used for a cross-session DA analysis based BCI calibration, were selected by pooling different combinations of existing sessions. How and when to adapt the classifier and which training data is optimal for classifier calibration and adaptation are still open questions in BCI research. Some researchers choose to adapt the classifier after every session, others periodically after a number of sessions but using the most recent session, whilst others apply online real-time and regular automated adaptation techniques (see [56] for short review). The approach we employed was thorough in terms of assessing which combination of past sessions maximized accuracy and was, additionally, robust and stable across multiple sessions—however, it may have been suboptimal as it was a heuristic search. Future work will involve developing a globally optimal solution to select the training data for more robust optimization as well as developing automated solutions for classifier updates and adaptation.

Additionally, as we did not use EEG data that was acquired during the training race attempts to update the classifier, we did not collect triggers from the BrainDriver platform to epoch the EEG. The challenge of determining a strategy to win the competition meant that the data and research focus was secondary. This may have been an oversight, as more game related data would have enabled a deeper analysis. Future work should focus on complete profiling of pilot affective states and race performance. Finally, this study presented a single subject whilst future work should involve comparing additional subjects competing within the same training regime.

## Conclusion

We described the long-term training of a pilot who has tetraplegia in preparation to compete at the Cybathlon Competition in 2019 and 2020. Training was undertaken in the pilot’s home, in hotel rooms, and at the race venues. All home-based training in 2020 was supported remotely by the team (i.e., due to the COVID 19 pandemic a non-expert at the pilot’s home assisted with cap preparation, etc., and the race team remotely monitored, coached, and calibrated performance). Our results demonstrate significant improvement in performance as a result of our user training strategy, BCI approach and optimisation of the BCI parameters. We have demonstrated that applying multiple binary classifiers along with additional post-processing modules and training with multiple neurogaming technologies is effective at improving the capacity of a user who has tetraplegia to control a virtual avatar without movement, directly via brain activity, and to maximise that control ability to continue to reduce race times and achieve state-of-the art performances for the challenge. Our pilot has developed into a BCI expert, even though he has tetraplegia for 37 years, as demonstrated by consistently achieving accuracies above 90% and competitive race times outside the competition days. We did however observe that performance was significantly impacted by changes in cognitive state, possibly due to heightened arousal arising from competition day pressure. We conclude that relax and testing state modulations are not stable and helping the pilot to maintain consistent relax/resting states is of critical importance to ensure race day performances are consistent with best training day performance. This should be supplemented by adaptive BCI strategies that can autonomously adjust to cognitive state changes to maintain performance. However, maintenance of cognitive state stability is likely to be the most important criteria for success at the Cybathlon championship for people with disabilities. We will focus on this and plan to compete again at the CYBATHLON Edition in 2024.

## Supplementary Information

**Additional file 1: Video S1.** NeuroSensi game - Dublin 2019.

**Additional file 2: Video S2.** BrainDriver game - Graz 2019.

**Additional file 3: Video S3.** BrainDriver and Triad games - ISRC, Derry 2020.

**Additional file 4: Video S4.** Triad games - ISRC, Derry 2020.

## Data Availability

The datasets used and/or analysed during the current study are available from the corresponding author on reasonable request.

## Abbreviations

BCI: Brain–computer interface
ERD: Event-related de-synchronization
ERS: Event-related synchronization
SMR: Sensorimotor rhythms
EEG: Electroencephalography
UDP: User datagram protocol
FBCSP: Filter-bank common spatial patterns
MI: Mutual information
DA: Decoding accuracy
CSP: Common spatial patterns
RLDA: Regularized linear discriminant analysis
LDA: Linear discriminant analysis
TSD: Time-varying signed distance
sLoreta: Standardized low resolution brain electromagnetic tomography
DB: Dead-band
DBB: Dead-band-break
CV: Cross-validation
STD: Standard deviation
PSD: Power spectral density
PIO: Pilot-induced oscillation
VR: Virtual reality
BAT: Boundary avoidance task
HRV: Heartrate variability
MBSR: Mindfulness-based stress reduction
ECG: Electrocardiography
